# A Meta-Analysis of Mortality in End-Stage Renal Disease Patients Receiving Implantable Cardioverter Defibrillators (ICDs)

**DOI:** 10.1371/journal.pone.0099418

**Published:** 2014-07-18

**Authors:** Tien-Hsing Chen, Hung-Ta Wo, Po-Cheng Chang, Chun-Chieh Wang, Ming-Shien Wen, Chung-Chuan Chou

**Affiliations:** 1 Department of Cardiology, Chang-Gung Memorial Hospital, Linko, Taiwan; 2 Department of Cardiology, Chang-Gung Memorial Hospital, Xiamen, China, Chang Gung University College of Medicine, Kaohsiung, Taiwan; Medical University Innsbruck, Austria

## Abstract

Data on the effectiveness of implantable implantable cardioverter defibrillators (ICDs) with respect to reducing mortality in patients with chronic kidney disease (CKD) and end-stage renal disease (ESRD) are lacking. The purpose of this meta-analysis was to compare the mortality of patients with ESRD who have received and not received an ICD. A search was conducted on January 31, 2013 of Medline, Cochrane, EMBASE, and Google Scholar. Studies were selected for inclusion based on the following criteria. 1) Randomized controlled trial. 2) ESRD patients with heart failure. 3) Device therapy (ICD, CRT-defibrillator [CRT-D]) used to treat heart failure. 4) Primary outcome is survival analysis. 5) Retrospective study if survival analysis was performed. The primary outcome was overall survival (OS), and the secondary outcome was 2-year survival. Odds ratios (ORs) with 95% confidence intervals (CI) were calculated, and a χ^2^-based test of homogeneity was performed. Three studies were included in the analysis. The combined OR for OS was 2.245 (95% CI 1.871 to 2.685, P<0.001), indicating that patients with an ICD had a significantly higher OS than those without an ICD. The combined OR for 2-year survival was 2.312 (95% CI 1.921 to 2.784, P<0.001), indicating that patients with an ICD had a significantly higher 2-year survival rate than those without an ICD. The use of ICD in patients with ESRD is associated with an increase in the OS and the 2-year survival rate.

## Introduction

The number of patients with end-stage renal disease (ESRD) receiving dialysis is increasing worldwide. It is estimated that in the United States alone more than 2 million people will be receiving dialysis by 2020 [1]. Patients receiving dialysis have approximately 8-fold greater all-cause mortality as compared to the general population, with cardiovascular disease accounting for approximately 43% of the mortality [1], [2]. Individuals with chronic kidney disease (CKD) are at a markedly increased risk of death from cardiovascular causes, including sudden cardiac death (SCD) due to arrhythmias [3], [4].

Implantable implantable cardioverter defibrillators (ICDs) have been shown to reduce mortality and the risk of SCD in patients with severe heart failure as a result of ischemic and nonischemic cardiomyopathy, and in patients with arrhythmias [5]–[8]. However, data on the effectiveness of ICDs in patients with CKD and ESRD are lacking, and sometimes conflicting [9]–[14]. This is in part because patients with renal disease were often excluded from ICD studies [15], [16]. It has been proposed that the survival advantage of ICDs in patients with renal disease as suggested by some studies may be negated as a result of comorbidities such as anemia, diabetes, and hypertension in these patients [15].

Tompkins et al. [17] reported that bleeding and ICD device-related complications were significantly more common in patients with ESRD. Alsheikh-Ali et al. [18] categorized patients by New York Heart Association (NYHA) class and estimated glomerular filtration rate (eGFR). The analysis suggested that the benefits of ICDs in patients with more advanced disease may be limited by the greater frequency of deaths due to causes other than arrhythmias. Bilchick et al. [19] found that CKD was associated with increased mortality rate in patients undergoing ICD implantation for the primary prevention of SCD (hazard ratio [HR] = 2.33). Similarly, a meta-analysis performed by Korantzopoulos et al. [20] in 2009 suggested that CKD is associated with increased mortality in patients who receive ICD therapy.

Given the increase in mortality rate of patients with CKD and ESRD, and the paucity of data regarding the outcome of ICD implantation in this group of patients, further investigation is warranted. The purpose of this study was to perform a meta-analysis to compare the mortality of ESRD patients receiving device therapy (ICD) with those who did not received device therapy.

## Methods

### Literature Search Strategy

A search was conducted of Medline, Cochrane, EMBASE, and Google Scholar using combinations of the search terms: chronic kidney disease, end-stage renal disease, dialysis, heart failure, mortality, survival, device therapy, implantable cardioverter-defibrillator/ICD, cardiac resynchronization therapy defibrillator/CRT-D. The search date was January 31, 2013. Each publication was carefully examined, including the names of all authors, to avoid duplication of data.

### Selection criteria

Studies were selected for analysis based on the following inclusion criteria. 1) Randomized controlled trial. 2) ESRD patients with heart failure. 3) Device therapy (ICD, CRT-defibrillator [CRT-D]) used to treat heart failure. 4) Primary outcome is survival analysis. 5) Retrospective study if the survival analysis was performed. Exclusion criteria for this analysis were as follows. 1) Study participants were not ESRD patients. 2) The study was not designed for ESRD patients with/without device therapy. 3) Studies that investigated if ESRD is risk factor/predictor of the prognosis for heart failure patients with device therapy (ICD, CRT-D). 4) Survival rate was not part of the analysis.

### Data extraction

Studies were identified by two independent reviewers using the aforementioned search strategy. A third reviewer was consulted when there was uncertainty regarding eligibility.

The following data were extracted from studies that met the inclusion criteria: name of the first author, year of publication, study design, number of participants in each treatment group, participants' age and gender, overall survival (OS) rate, median OS time, 2-year survival rate, rate of comorbidities related to heart failure

### Data analysis

The primary outcome was OS, and the secondary outcome was 2-year survival rate. The primary outcome, OS was used to evaluate treatment efficacy. Odds ratios (ORs) with 95% confidence intervals (CI) were calculated for binary outcomes and compared between patients with and without device therapy. A χ^2^-based test of homogeneity was performed, and the inconsistency index (I^2^) statistic was determined. If I^2^ was >50% or >75%, the trials were considered to be heterogeneous or highly heterogeneous, respectively. An I^2^<25% indicated homogeneity among the studies. When heterogeneity existed between studies (I^2^>50%) a random-effects model was calculated. Otherwise, fixed-effects models were calculated. Pooled summary statistics for ORs of the individual studies were reported. Sensitivity analysis was performed based on the leave-one-out approach. Publication bias analysis was not performed because the number of studies was too few to detect an asymmetric funnel [21]. All analyses were performed using Comprehensive Meta-Analysis statistical software, version 2.0 (Biostat, Englewood, NJ). A value of P<0.05 was considered to indicate statistical significance.

## Results

### Literature search

**Figure 1:**
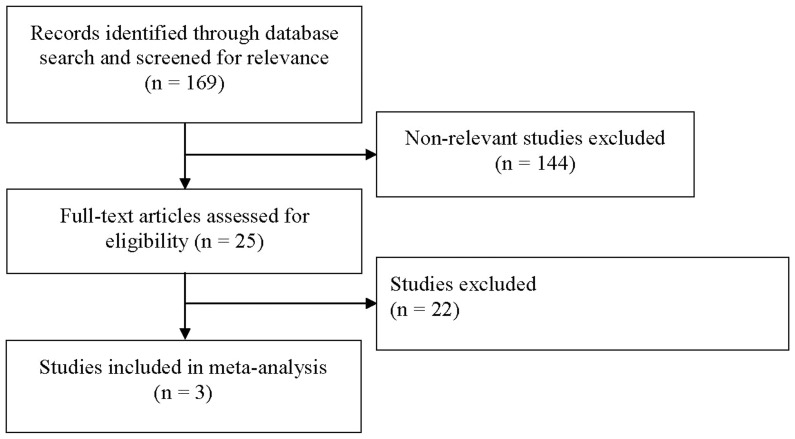
Flow diagram of study selection.

After applying the inclusion and exclusion criteria, a total of 3 studies were included in this meta-analysis [22]–[24]. A flowchart of the study selection is shown in Figure 1. The 3 studies included in the meta-analysis are summarized in Table 1. Two studies included only ESRD patients [22], [24], whereas one study included both CKD and ESRD patients [23]. For the purposes of this analysis, only data of ESRD patients from the study by Khan et al. [23] were used in the analysis.

**Table 1:** Summary of the 3 studies included in the meta-analysis.

1st Author	Year of Publication	Study Type	Group	Number of Patients	Age (y)	Sex (male)	OS rate	Median OS time	2-year Survival Rate	Rate of heart failure-related comorbidity
Hiremath[22]	2010	Retrospective	ICD	50	70.3±10.0	84%	60%	8 years	84%	ND
			Non-ICD	50	69.9±9.7	84%	42%	3.1 years	60%	ND
Khan* [23]	2010	Retrospective	ICD	14	64±12	93%	35.7%	ND	55%	100%
			Non-ICD	31	63±15	68%	32.3%	ND	42%	100%
Herzog[24]	2005	Retrospective	ICD	460	63.1±13.1	57%	51%	ND	53%	60.4%
			Non-ICD	5582	63.1±14.2	46%	31%	ND	33%	55.8%

Age data are presented as mean ± standard deviation.

OS, overall survival; ND, not derived.

*Only data of patients with end-stage renal disease were used in the analysis.

### Study characteristics and clinical outcomes

**Figure 2:**
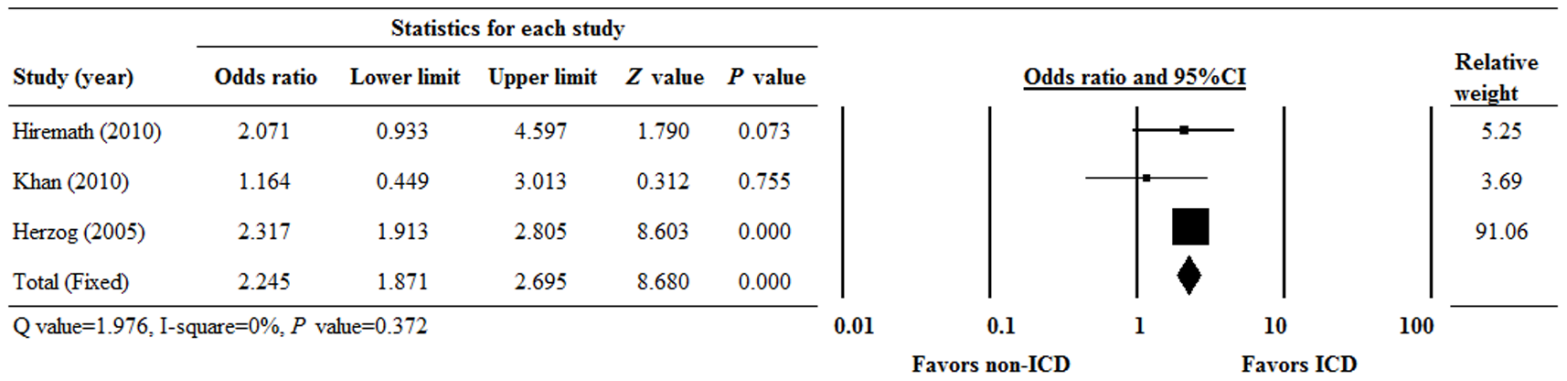
Forest plot of odds ratios (ORs) with 95% confidence intervals (CIs) for overall survival (OS) of the included studies. P<0.05 indicates a statistically significant difference.

The ORs for OS of the 3 studies ranged from 1.164 to 2.317 (Fig. 2). There was homogeneity in the combined OR among the 3 studies (Q = 1.976, I^2^ = 0%, P = 0.372); therefore a fixed-effects model of analysis was used. Examination of the combined OR revealed a significant difference between ICD and no-ICD therapy. The combined OR was 2.245 (95% CI 1.871 to 2.685, P<0.001), indicating that patients with an ICD had a significantly higher OS than those without ICD therapy.

**Figure 3:**
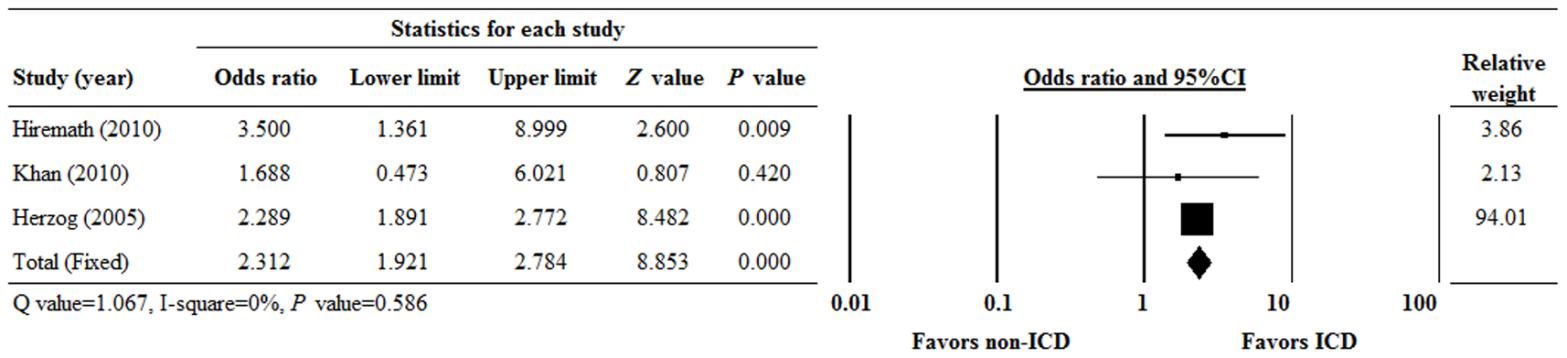
Forest plot of odds ratios (ORs) with 95% confidence intervals (CIs) for 2-year survival of the included studies. P<0.05 indicates a statistically significant difference.

The ORs for the 2-year survival rate of the 3 studies ranged from 1.688 to 3.500 (Fig. 3). There was homogeneity in the 2-year survival rate between the studies when the data were pooled for analysis (Q = 1.067, I^2^ = 0.00%, P = 0.586); therefore a fixed-effects model of analysis was used. Examination of the combined OR revealed a significant difference between ICD and no-ICD therapy. The combined OR was 2.312 (95% CI 1.921 to 2.784, P<0.001), indicating that patients with an ICD had a significantly higher 2-year survival rate than those without ICD therapy.

**Figure 4:**
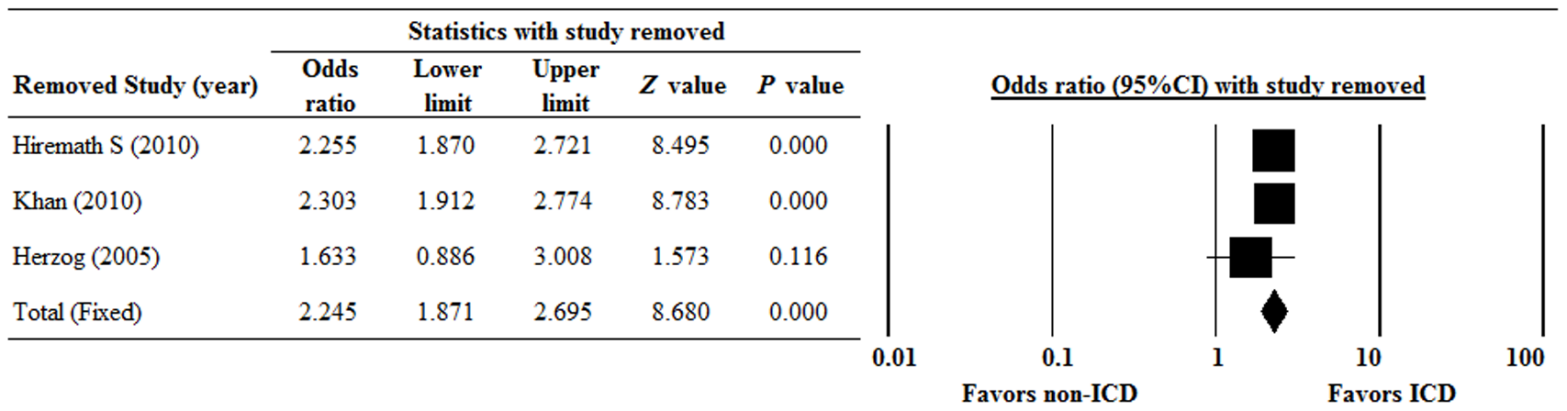
Sensitivity analysis for the influence of individual studies on pooled estimates by the leave-one-out approach for overall survival (OS). Data are presented as odds ratio (OR) with the 95% confidence interval (CI). P<0.05 indicates a statistically significant difference.

Figure 4 shows the results of the meta-analysis of OS with one study removed in turn. The results indicate that the direction and magnitude of the combined estimates did not have a large variation. This finding indicates that the results of the meta-analysis exhibits good reliability.

## Discussion

The results of this meta-analysis indicate that the use of ICD in patients with ESRD is associated with an increase in the OS and the 2-year survival rate.

Data from prospective, randomized studies examining the effect of ICD therapy in patients with CKD/ESRD are lacking, and thus there is no general consensus on the use of device therapy in these patients. An ongoing trial (ICD2 trail) is randomizing dialysis patients, regardless of left ventricular function, to receive ICD therapy or not; however, study results are not expected until 2017 [25]. Most small retrospective studies have failed to show that patients with CKD or ESRD derive any survival benefit from ICD implantation [9], [11]–[14]. A subgroup analysis of data from the MADIT-II study showed a survival benefit of ICD implantation in patients with an eGFR >35 mL/min, but not in those in which the eGFR was ≤35 mL/min [9], [10]. Studies have reported a 1-year survival of patients with CKD with ICD implantation of 61% [12] and median survival of 6.3 years [14], and a median survival of ESRD patients with an ICD of 1.1 to 3.2 years [12], [13].

Three studies were included in this meta-analysis. Herzog et al. [24] examined dialysis patients hospitalized from 1996 to 2001 for ventricular fibrillation/cardiac arrest who received ICD implantation within 30 day of admission. In the cohort, there were 460 patients (7.6%) who received ICD implantation and 5,582 (92.4%) that did not. The estimated 1-, 2-, 3-, 4-, and 5-year survival rates in the ICD group were 71%, 53%, 36%, 25%, and 22%, respectively, and in the no-ICD group were 49%, 33%, 23%, 16%, and 12% (P<0.0001). Analysis of the data showed that ICD implantation was independently associated with a 42% reduction in the risk of death (relative risk [RR] = 0.58). The authors concluded that in addition to the improvement in survival, ICD therapy was underutilized in this population. Khan et al. [23] studied 78 patients with moderate to severe CKD (45 patients with ESRD) with a left ventricular ejection fraction (LVEF) ≤35%, of whom 32 had an ICD, for an average follow-up of 2.7±2.3 years. In the group receiving dialysis (n = 45), ICD placement did not impact survival. In the patients with CKD who were not receiving dialysis (n = 33), survival was significantly better in patients with an ICD (2-year survival 80% vs. 54%, P = 0.027) after adjustment for sex, race, GFR, digoxin use, and presence of coronary disease, heart failure, or hypertension (OR = 0.23). Hiremath et al. [22] compared the outcomes of 50 patients with ESRD who had received ICD implantation with 50 patients with ESRD who did not have ICDs. The mean LVEF was similar between the 2 groups (approximately 29%). Median OS in the full cohort was 4.7 years with 20 deaths in the ICD group and 29 deaths in the no-ICD group. The median survival in the ICD group was 8.0 years, and 3.1 years in the no-ICD group. The multivariable analysis indicated that all-cause mortality was significantly less in the ICD group than in the no-ICD group (HR = 0.40).

The benefits of ICDs have been shown to be reduced in patients with advanced renal disease [9], [10], [26]. Furthermore, the complication rate of ICD implantation is higher in patients with ESRD than in patients without ESRD [27]-[29]. Patients with CKD have increased mortality from non-cardiac causes, cardiac non-SCD, SCD, and infections and while ICD implantation may decrease the risk of SCD it will not affect the risk of death from non-cardiac causes such as infection, and there is increased risk of complications from device placement. The risk of SCD increases as renal function deteriorates, and this increase in risk is multifactorial in origin. The incidences of coronary artery disease, left ventricular hypertrophy, and left ventricular dysfunction are all increased in patients with ESRD. In addition, dialysis can lead to the development of interstitial fibrosis, endothelial dysfunction, and atheroma formation, which all can worsen the aforementioned conditions. The above highlight the competing causes of death in patients with CKD; conditions that are not affected by ICD placement.

The difference in survival between patients receiving dialysis and those not receiving dialysis as reported by Khan et al. [23] may be because in CKD patients ventricular arrhythmias can be terminated with ICD therapy [30]. In patients receiving dialysis, however, comorbidities which are not affected by ICD therapy may be present [31], [32]. It has also been suggested that the defibrillation threshold may be increased in patients with ESRD, and thus optimal conversion of arrhythmias may not occur [33]. Despite the use of an ICD, the OS of patients with CKD is lower as compared to patients with normal kidney function [34]. On the other hand, CKD patients with an ICD still benefit from improved survival with ICD placement. For example, Amin et al. [35] showed in patients with stage 1 and 2 CKD, ICD implantation reduces mortality; however, in more advanced stages of CKD the benefit is less significant and age dependent. The authors attribute this finding to the fact that patients with more advanced CKD having a higher procedural risk and decreased life expectancy. When average procedural mortality was taken into account, the authors found that ICD implantation is favored at <80 years of age for stage 3 CKD, at <75 years of age for stage 4 CKD, and at <65 years of age for ESRD.

ICD therapy appears to be underutilized in patients with CKD, although patients with ESRD are at high risk for ventricular arrhythmias and SCD. Herzog et al. [24] reported a 42% reduction in overall death risk in dialysis patients, yet only 8% of eligible patients received an ICD. Other data [1] and studies [36] have also indicated that the use of ICD therapy in patients with CKD and ESRD is low. Therapies such as aspirin, beta blockers, and angiotensin converting enzyme inhibitors are used less frequently in patients with more severe renal failure [37], and thus physicians may be less likely to use other therapies (i.e., ICD) as well. There is also the concern of increased complications of ICDs in patients with renal failure [17]. Finally, as previously discussed; there is lack of data from well-designed clinical studies for this group of patients. Interestingly, the 2013 American College of Cardiology Foundation (ACCF) guidelines for the use of implantable ICDs and CRT include patients with CKD and ESRD [38].

The primary limitation of this meta-analysis is the small number of included studies. However, the inclusion criteria were strict by design to include only studies that were high quality and relevant to addressing the research question. In addition, only patients with ESRD were included. It remains to be determined if the results are also applicable to patients with CKD, but not ESRD.

## Conclusions

In conclusion, the results of this meta-analysis indicate that the use of an ICD in patients with ESRD is associated with an increase in the OS and the 2-year survival rate. Based on these results, the use of ICD therapy in these patients is warranted.

## Supporting Information

Checklist S1 PRISMA Checklist.
(DOC)
